# A Supervised ML Applied Classification Model for Brain Tumors MRI

**DOI:** 10.3389/fphar.2022.884495

**Published:** 2022-04-08

**Authors:** Zhengyu Yu, Qinghu He, Jichang Yang, Min Luo

**Affiliations:** ^1^ Department of Nephrology, The Second Xiangya Hospital, Central South University, Changsha, China; ^2^ Faculty of Engneering and IT, University of Technology Sydney, Sydney, NSW, Australia; ^3^ Department of Rehabilitation Medicine and Health Care, Hunan University of Medicine, Huaihua, China

**Keywords:** brain tumor, magnetic resonance imaging, machine learning algorithms, classification, automation

## Abstract

Brain Tumor originates from abnormal cells, which is developed uncontrollably. Magnetic resonance imaging (MRI) is developed to generate high-quality images and provide extensive medical research information. The machine learning algorithms can improve the diagnostic value of MRI to obtain automation and accurate classification of MRI. In this research, we propose a supervised machine learning applied training and testing model to classify and analyze the features of brain tumors MRI in the performance of accuracy, precision, sensitivity and F1 score. The result presents that more than 95% accuracy is obtained in this model. It can be used to classify features more accurate than other existing methods.

## Introduction

In the human body, the brain is a complex organ. When brain tumors originate, uncontrolled cell division occurs in an abnormal series of cells forms in the brain (Logeswari and Karnan, 2010). That abnormal series of cells will destroy healthy cells and influent the general activity of the brain. Benign tumors and malignant tumors Brain are two classifications of brain tumors. Benign tumors grow slowly and originate in the brain; They are considered non-progressive or non-cancerous. Benign tumors cannot extend to any other organs inside the body. In contrast, malignant tumors are progressive and cancerous. They grow unexpectedly in an indeterminate manner. Primary malignant tumors can grow themselves. In addition, malignant tumors also can grow in other organs inside the body and spread to the brain.

**FIGURE 1:**
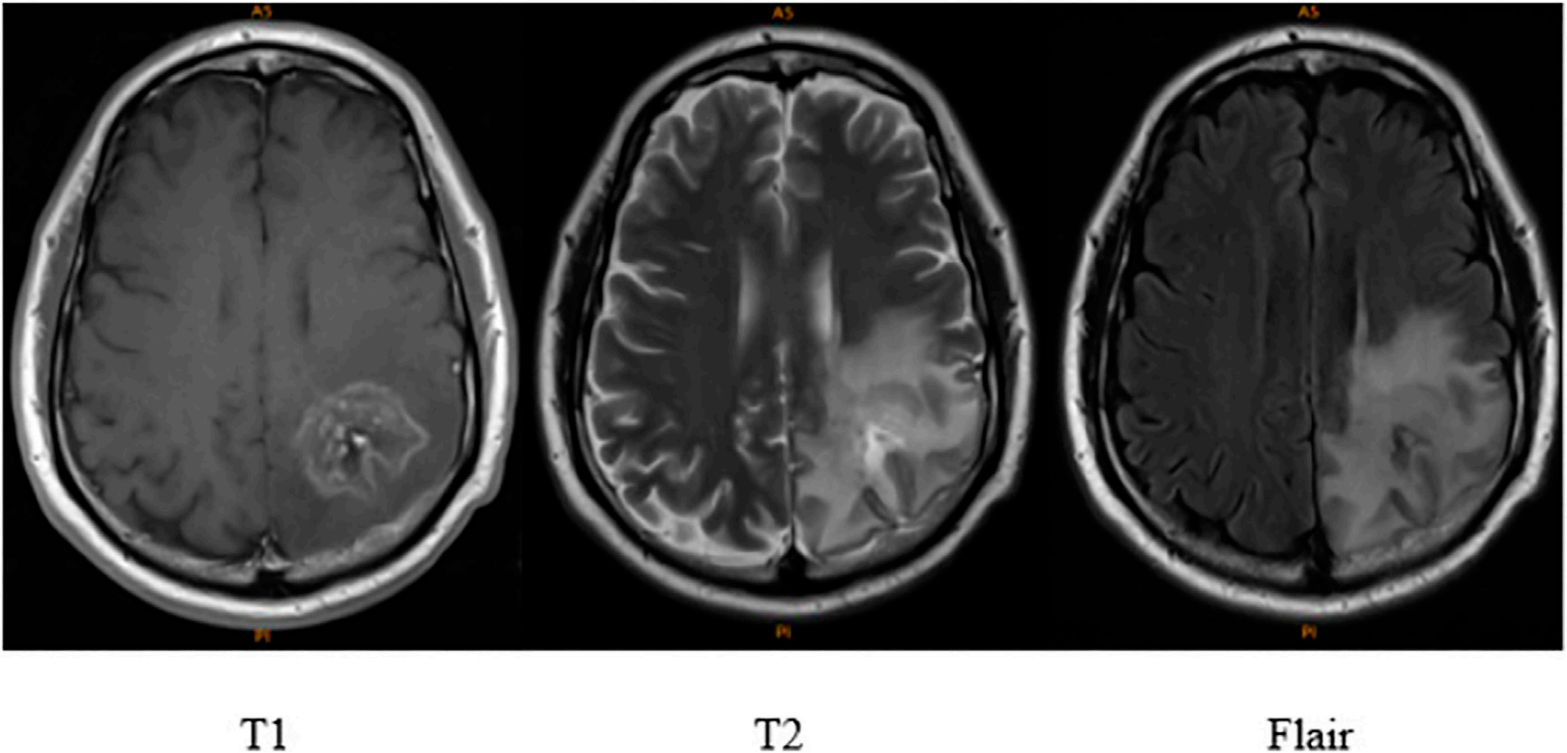
Comparison of T1, T2 and flair of brain tumors MRI (Clark et al., 2013; Scarpace et al., 2022).

MRI is an imaging technology that can generate high-quality images of human anatomy. MRI provides extensive information for medical diagnosis and research (Zhang et al., 2011). The automation and accurate classification of MRI images has dramatically improved the diagnostic value of MRI (Scapaticci et al., 2012). However, one type of MRI cannot provide full details for brain tumours that contain many different tissues (Sudharani et al., 2016). Different weighted images are combined to develop the image segmentation of brain tumors. Three weighted MRI images (T1, T2, and FLAIR, in Figure 1) are used for image segmentation of the skull on different axial slices (Vannier et al., 1988; Clark et al., 1994; Dou et al., 2007).

As one of the best imaging methods, researchers use MRI to analyze the progression of a brain tumor during the stages of detection and treatment. As MRI generates high resolution, brain structure information, such as brain tissue abnormalities, is detailed. Therefore, MRI significantly influences automatic analysis for medical images (Zacharaki et al., 2009; Litjens et al., 2017). Since medical images can be scanned and loaded into a computer, researchers have proposed different automated methods of observation and classification for brain tumor by exploiting brain MRI images (Litjens et al., 2017).

Recently, two categories of research have been proposed. First is unsupervised classification, such as fuzzy c-means and self-organization feature maps (Ibrahim et al., 2013). Second is supervised classification, such as K Nearest Neighbours (KNN) and Support Vector Machine (SVM) (Cocosco et al., 2003; Chaplot et al., 2006). According to the results in classification accuracy, the performance of supervised classification is better than unsupervised classification (Zhang and Wu, 2008; Ibrahim et al., 2013). Nevertheless, most of the classification accuracy is less than 95% (Yeh and Fu, 2008). In the past decades, SVM and Neural Network (NN) become popular due to the outstanding performance for detecting and classifying brain tumors (Ibrahim et al., 2013). Recently, deep learning methods have established novel modeling in machine learning. Complex relationships can be displayed effectively without the need for many nodes by deep architectures, such as SVM and KNN. In this case, they have rapidly developed into the most advanced technologies in various health research fields (such as medical image analysis, medical informatics, and bioinformatics) (Pan et al., 2015; Ravì et al., 2016; Litjens et al., 2017).

## Materials and Methods

**FIGURE 2:**
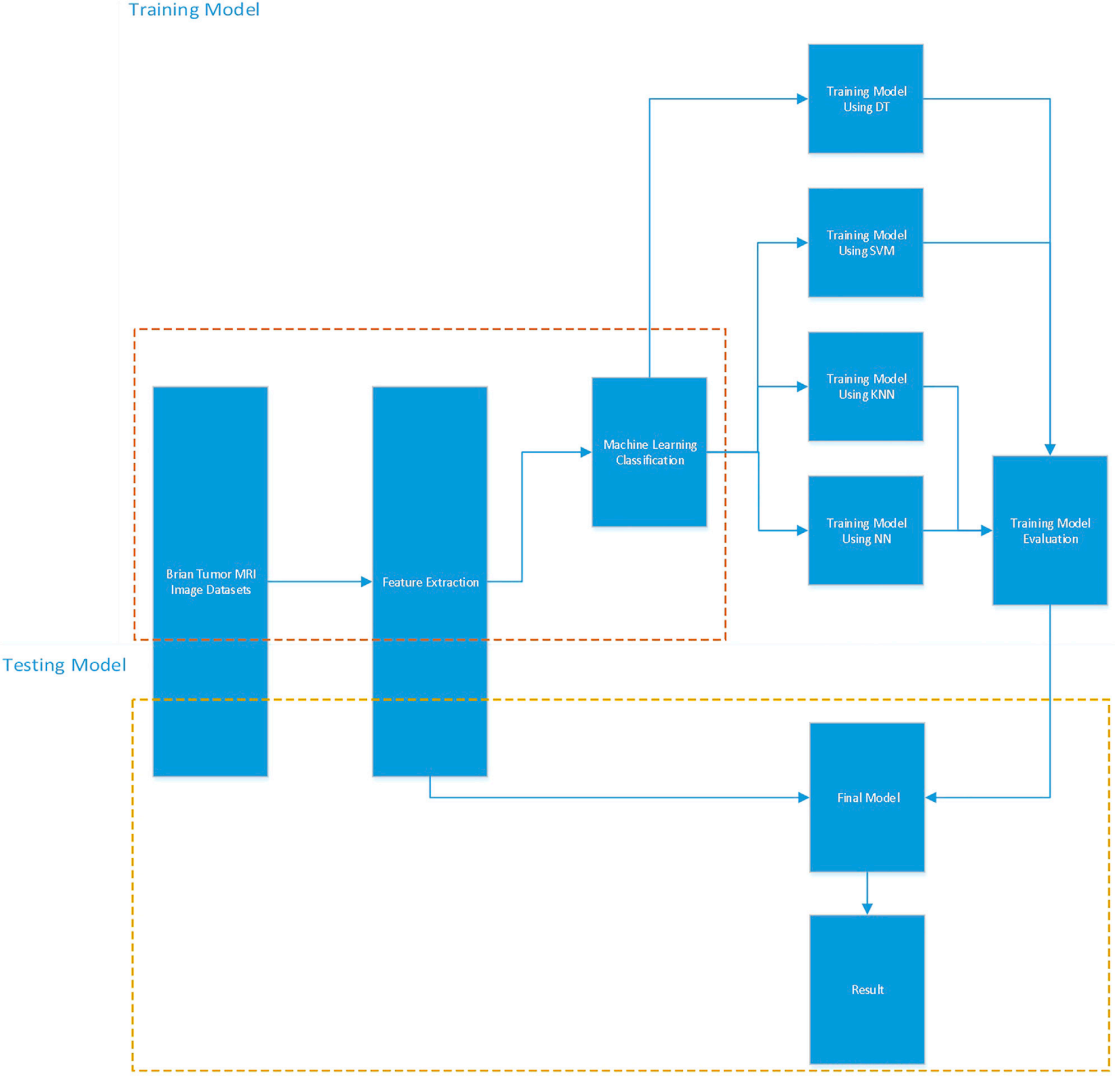
Workflow diagram for training model and testing model.

Supervised machine learning algorithms applied classification method is proposed to classify whether the cysts are detected from the MRI of brain tumors. Figure 2 illustrates the workflow diagram for the training and testing models of the classification method. The process is summarised below:

1) Extract datasets of Brain tumors MRI images. The datasets are from the Repository of Molecular Brain Neoplasia Data (REMBRANDT) in this research (Clark et al., 2013; Scarpace et al., 2022).
2) Extract features. Table 1 presents that there are 30 features extracted from brain tumors MRI, including 21 categorical features and 9 numerical features. Feature 8 is selected as a target feature; The rest are selected as attributes.
3) Machine learning algorithm classification comparison. Supervised machine learning algorithms applied classification methods, such as Decision Tree (DT), SVM, KNN and NN have been compared to estimate the performance for each training model. Cross-validations are computed on different folds to avoid overfitting. 80% of the datasets are used for training model. The result indicates that the model using DT is the most accurate.
4) The testing model is evaluated by using 20% of the datasets; in this stage, feature 8 is also selected as a target feature; the rest of the features are selected as attributes. The results present that the performance of the DT model with 30 cross-validation folds is the best.
5) After the final model has been evaluated, the result is predicted that the accuracy of the final model is 95.9%.

**TABLE 1:** Data features extracted from brain tumors MRI.

Number	Features	Type	Number	Features	Type
1	Tumor Location	Categorical	2	Side of Tumor Epicenter	Categorical
3	Eloquent Brain	Categorical	4	Enhancement	Categorical
				Quality	
5	Proportion	Numerical	6	Proportion nCET	Numerical
	Enhancing				
7	Proportion	Numerical	8	Cyst(s)	Categorical
	Necrosis				
9	Multifocal or Multicentric	Categorical	10	T1/FLAIR RATIO	Categorical
11	Thickness of enhancing margin	Categorical	12	Definition of the enhancing margin	Categorical
13	Definition of the non-enhancing	Categorical	14	Proportion of Edema	Numerical
	margin				
15	Edema Crosses	Categorical	16	Hemorrhage	Categorical
	Midline				
17	Diffusion	Categorical	18	Pial invasion	Categorical
19	Ependymal invasion	Categorical	20	Cortical involvement	Categorical
21	Deep WM invasion	Categorical	22	nCET tumor	Categorical
				Crosses Midline	
23	Enhancing tumor	Categorical	24	Satellites	Categorical
	Crosses Midline				
25	Calvarial remodeling	Categorical	26	Extent of resection of enhancing	Numerical
				tumor	
27	Extent resection of nCET	Numerical	28	Extent resection of vasogenic edema	Numerical
29 and 30	Lesion Size	Numerical			

### Datasets

The dataset we used for the research is REMBRANDT (Scarpace et al., 2022). It is accessed from The Cancer Imaging Archive (TCIA) database (Clark et al., 2013). REMBRANDT is purposed to explore the link between the data from genomic characterization and clinical information. and clinical information. REMBRANDT consists of pre-surgical MRI for 130 patients, including 174 studies, 1,483 series, and 110,020 images. Table 1 presents 30 extracted features from brain tumors MRI, including 21 categorical features and 9 numerical features.

### Training Algorithms Methods

The DT classifier is a supervised machine learning technique to make decisions in a multistage way. The decision tree’s fundamental concept includes spreading a complicated decision into a group of more straightforward decisions. The result from this technique could be similar to the intended desired result (Hastie et al., 2009).

The DT technique is a widely used data mining methodology to classify multiple covariates or predict a target variable by algorithms. Branda-like segments are classified *via* decision tree to consist of an inverted tree containing leaf node, interal node and the root node. The decision tree algorithm can efficiently determine complex and large data sets as its non-parametric structure. The data for the study is separated for training and validation when the data set size is too large. The training data sets are built for the decision tree model, whereas the validation data sets are built to approach the optimal final solution by appropriate tree size (Boser et al., 1992; Song and Lu, 2015).

SVM is a commonly used machine learning methodology that classifies data mining problems by its relative flexibility and simplicity (Hearst et al., 1998). SVMs have been processed in a wide variety of biomedical applications. For instance, SVM can help automatically classify microarray gene data sets, where the gene expression profile can be examined if they are derived from peripheral fluid or a tumour sample for the result of diagnosis or prognosis. In brain diseases search, SVMs are usually applied by multivoxel pattern analysis due to the low possibility of overfitting when processing images with high dimensions. Recently, SVMs have been developed to predict prognosis and diagnosis in brain disorders research (Orrù et al., 2012).

KNN is an effective and high-performance learning technique to classify and cluster data from a large scale in big data applications (Zhang et al., 2017). The original KNN technique typically set a value of K and select the nearest samples with the influential group. In selecting K nearest samples, KNN is calculated the similarity of all samples for training (Guo et al., 2003). This algorithm costs high memory of the computer and time to process extensive data. Nevertheless, KNN is one of the top techniques in data mining due to its significant performance (Deng et al., 2016).

NN has been introduced as a vital tool for classification in recent research. NN is non-linear and self-adaptive. It is flexible in a complex data environment and can alter itself based on data without explaining of classification functions (Cybenko, 1989; Hornik, 1991). Moreover, NN has the advantage of performing statistical analysis and establishing classification functions with their capability of estimating the probabilities of posterior (Richard and Lippmann, 1991; Zhang, 2000).

## Results and Discussion

The confusion matrix is applied to determine the accuracy, precision, sensitivity and F1 score for the performance of the classifier method. Table 2 shows the confusion matrix for the classifier method.

**TABLE 2:** Confusion matrix for the classifier method.

		Actual class	
		Positive class	Negative class
Predicted Class	Positive Class	True Positive (TP)	False Positive (FP)
	Negative Class	False Negative (FN)	True Negative (TN)

The accuracy, precision, sensitivity and F1 score are calculated by equations below:

$$\text{Accuracy}=\frac{TP+TN}{TP+TN+FP+FN}$$ (1)

$$\text{Precision}=\frac{TP}{TP+FP}$$ (2)

$$\text{Sensitivity}=\frac{TP}{TP+FN}$$ (3)

$$\text{F}1-\text{score}=\frac{2TP}{2TP+FP+FN}$$ (4)

### DT Classifier

**FIGURE 3:**
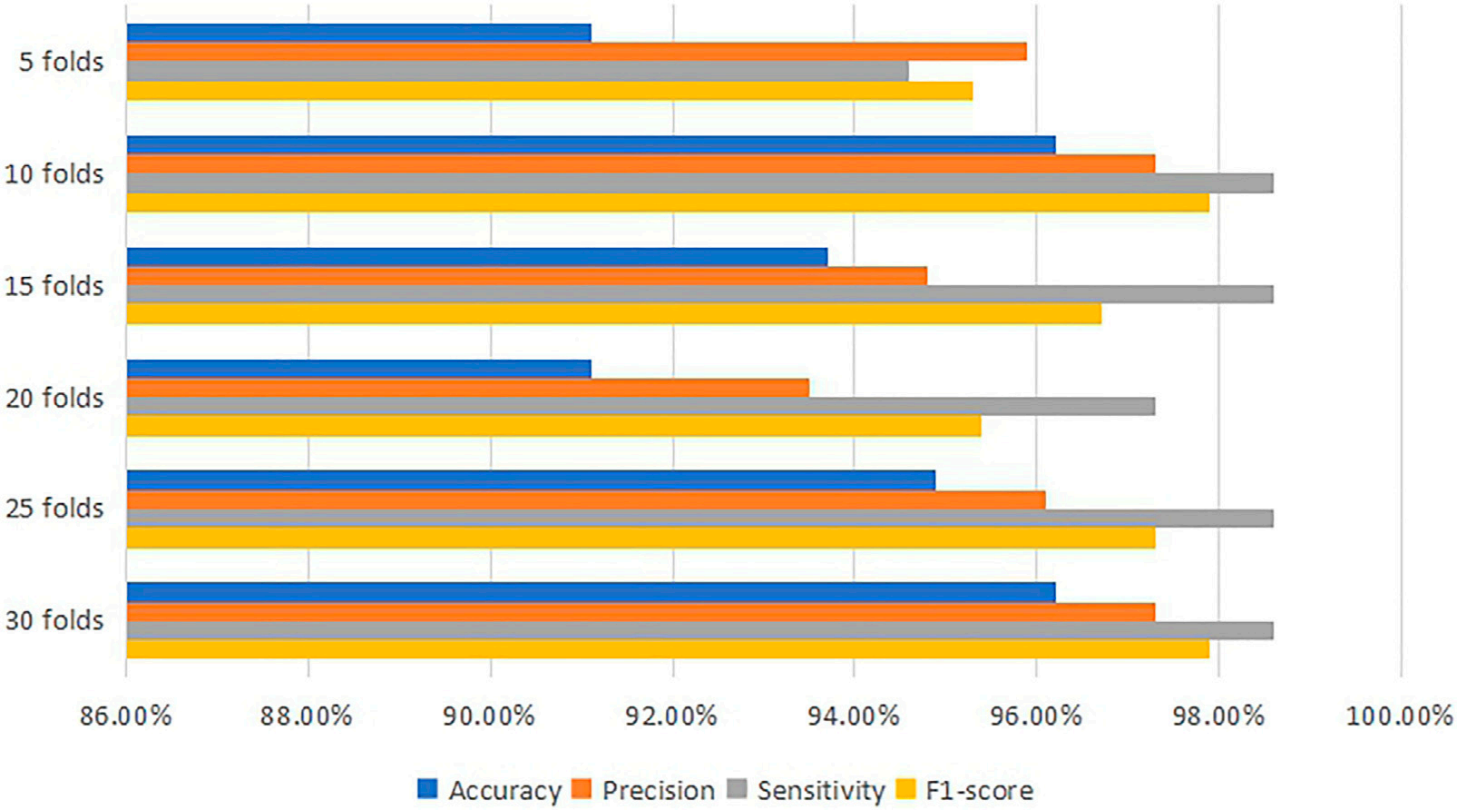
Comparison diagram for the performance of DT classifier.

After processing the training model, the machine learning classifier using DT algorithms indicates that the most accurate model is 96.2% at 30 folds cross-validation. Table 3 and Figure 3 present the value of accuracy, precision, sensitivity and F1-score for each fold cross-validation. At 30 folds cross-validation, 96.2% accuracy, 97.3% precision, 98.6% sensitivity and 97.9% F1-score are obtained.

**TABLE 3:** Performance of DT classifier.

	Accuracy (%)	Precision (%)	Sensitivity (%)	F1-Score (%)
5 folds	91.1	95.9	94.6	95.3
10 folds	94.2	96.3	97.6	96.9
15 folds	93.7	94.8	98.6	96.7
20 folds	91.1	93.5	97.3	95.4
25 folds	94.9	96.1	98.6	97.3
30 folds	96.2	97.3	98.6	97.9

### SVM Classifier

**FIGURE 4:**
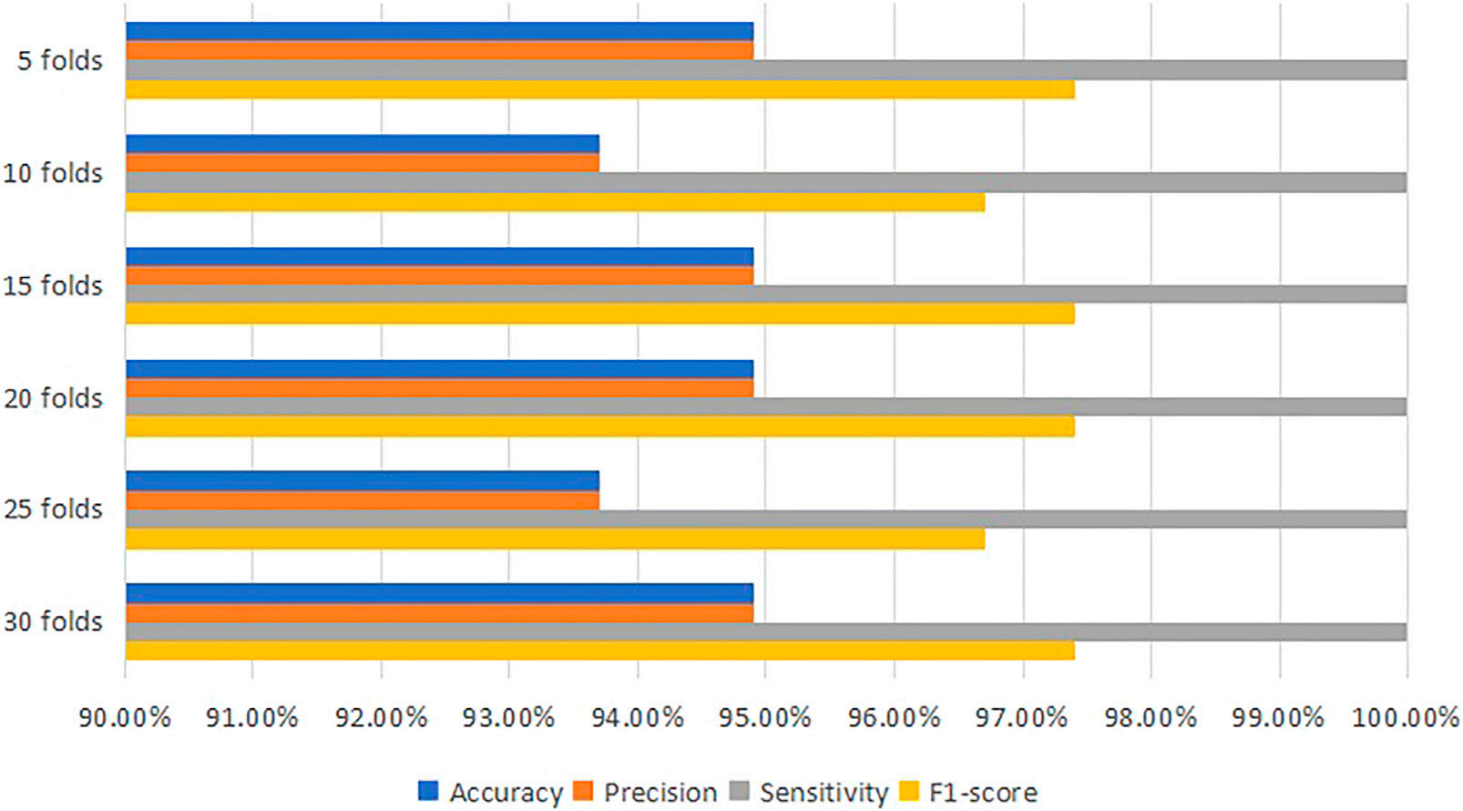
Comparison diagram for the performance of SVM classifier.

After the training model has been computed by SVM algorithms, Table 4 and Figure 4 indicate that the most accurate model is 94.9% at 5, 15, 20 and 30 folds cross-validation. They all obtain 94.9% accuracy, 94.9% precision, 100% sensitivity and 97.4% F1-score.

**TABLE 4:** Performance of SVM classifier.

	Accuracy (%)	Precision (%)	Sensitivity (%)	F1-Score (%)
5 folds	94.9	94.9	100	97.4
10 folds	93.7	93.7	100	96.7
15 folds	94.9	94.9	100	97.4
20 folds	94.9	94.9	100	97.4
25 folds	93.7	93.7	100	96.7
30 folds	94.9	94.9	100	97.4

### KNN Classifier

**FIGURE 5:**
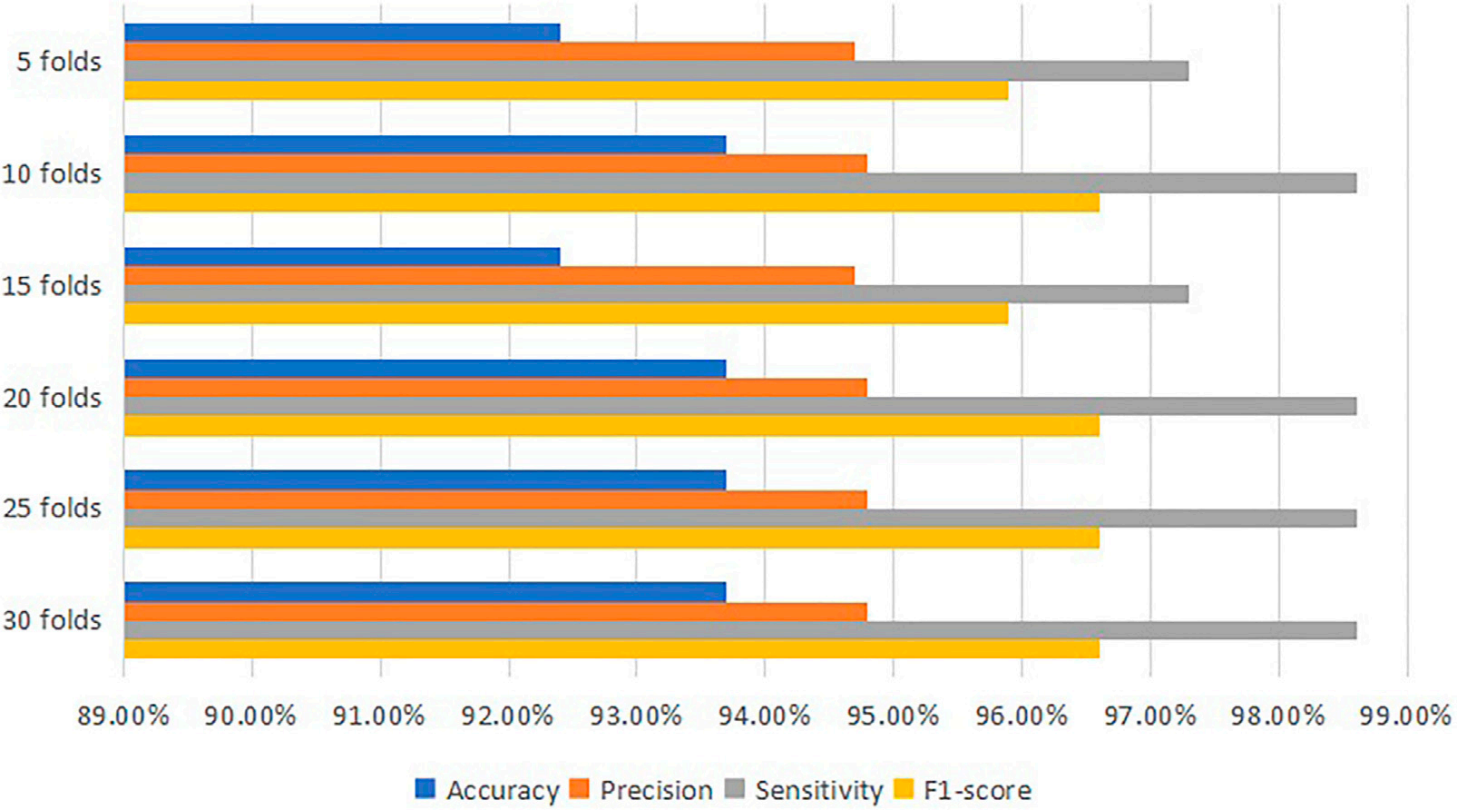
Comparison diagram for the performance of KNN classifier.

In this case, the training model has been processed by KNN Classifier, Table 5 and Figure 5 present that the most accurate model is 93.7% which are at 10 and 20 folds, 25 and 30 folds cross-validation. 93.7% accuracy, 94.8% precision, 98.6% sensitivity and 96.6% F1-score are obtained for all of them.

**TABLE 5:** Performance of KNN classifier.

	Accuracy (%)	Precision (%)	Sensitivity (%)	F1-Score (%)
5 folds	92.4	94.7	97.3	95.9
10 folds	93.7	94.8	98.6	96.6
15 folds	92.4	94.7	97.3	95.9
20 folds	93.7	94.8	98.6	96.6
25 folds	93.7	94.8	98.6	96.6
30 folds	93.7	94.8	98.6	96.6

### NN Classifier

**FIGURE 6:**
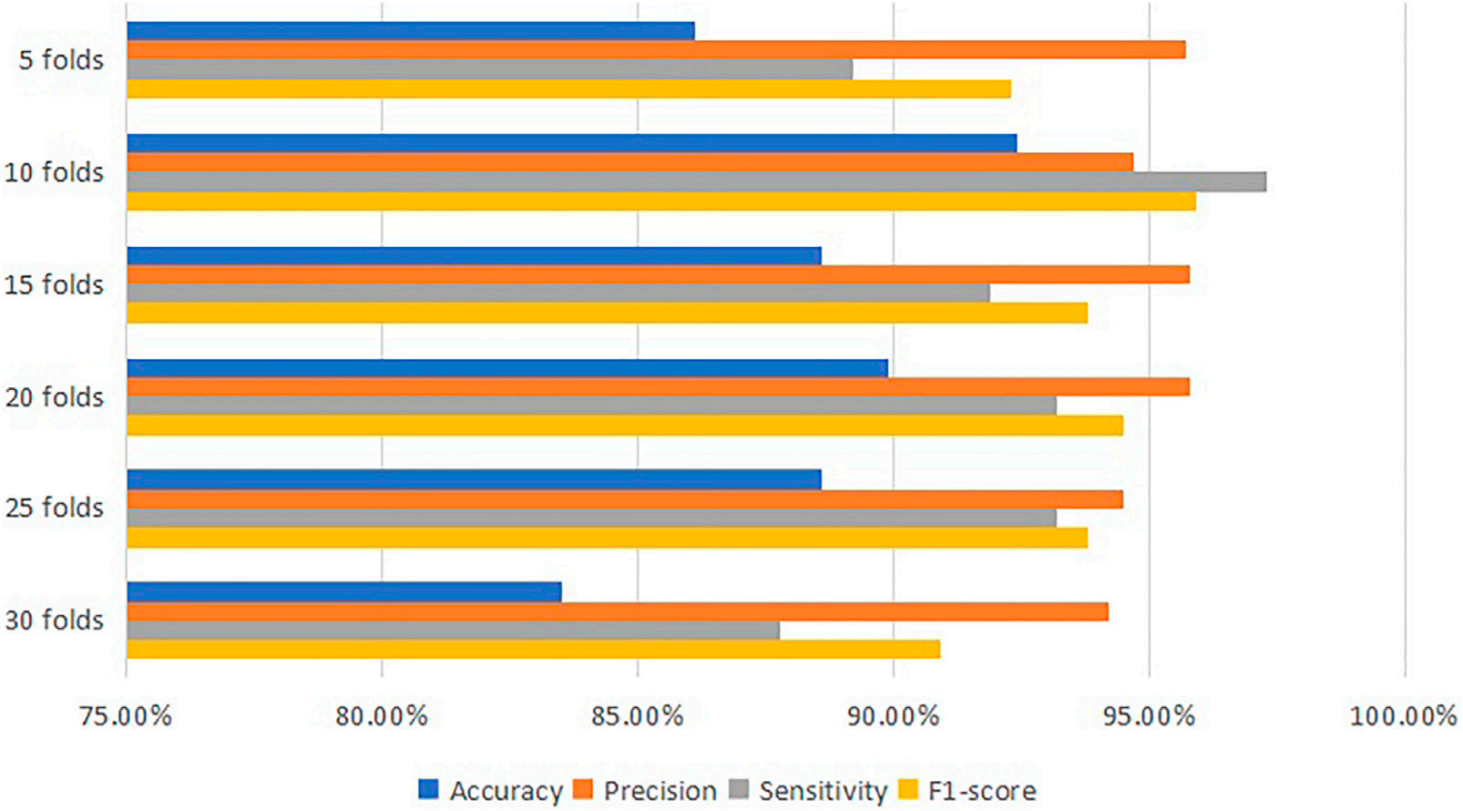
Comparison diagram for the performance of NN classifier.

Table 6 and Figure 6 are generated from the training model by NN classifier, they present that the most accurate model is 92.4% which is at 10 cross-validation with 94.7% precision, 97.3% sensitivity and 95.9%.

**TABLE 6:** Performance of NN classifier.

	Accuracy (%)	Precision (%)	Sensitivity (%)	F1-Score (%)
5 folds	86.1	95.7	89.2	92.3
10 folds	92.4	94.7	97.3	95.9
15 folds	88.6	95.8	91.9	93.8
20 folds	89.9	95.8	93.2	94.5
25 folds	88.6	94.5	93.2	93.8
30 folds	83.5	94.2	87.8	90.9

### Testing Model

All the classifiers are trained in the previous section. DT training model at 30 folds cross-validation with 96.2% accuracy is selected, which is the highest accurate model among the results. In this research, the testing model is used for evaluation with the rest of the datasets to verify the model’s performance.

As Table 7 presented, the accuracy of DT classifier at 30 folds cross-validation in the testing model is 95.9%. Although this is lower than the score in the training model due to the overfitting classification, it is still the best model with the highest performance.

**TABLE 7:** Performance of Testing model.

DT	Training (%)	Testing (%)
30 folds	96.2	95.9

## Conclusion

This article proposes a supervised machine learning applied classification model for brain tumors MRI. This model is developed to obtain higher classification performance of accuracy, precision, sensitivity and F1 score for the classification of features of brain tumors MRI. The optimized classification model with the most accurate result is developed by comparing with different supervised machine learning algorithms at different folds of cross-validation. After testing, the best performance of the model is obtained. This classification model can be used in other features of brain tumors MRI to obtain the most accurate result.

## Data Availability

The original contributions presented in the study are included in the article/Supplementary Material, further inquiries can be directed to the corresponding author.
